# Analysis of factors associated with cervical cancer and precancerous lesions

**DOI:** 10.1038/s41598-025-32215-3

**Published:** 2026-03-09

**Authors:** Danhong Hu, Yin Zhao, Mengnan Han, Weiwei Qian, Xiangqian Xu, Shaoqin Sheng

**Affiliations:** 1 Zhejiang Chinese Medical University, Hangzhou, 310016 Zhejiang China; 2 Department of Gynecology, Hangzhou First People’s Hospital, Hangzhou, 310016 Zhejiang China; 3 Department of Gynecology, Hangzhou Women’s Hospital/Hangzhou Maternity and Child Health Care Hospital, 369 Kunpeng Road, Hangzhou, 310016 Zhejiang China; 4 Department of Ultrasound, Ningbo No.2 Hospital, Ningbo, 315010 Zhejiang China; 5 Hangzhou Normal University, Hangzhou, 311121 Zhejiang China

**Keywords:** Cancer of the cervix, HPV infection and CIN, Epidemiology of GYN cancers, Cancer epidemiology, Gynaecological cancer, Tumour virus infections, Gynaecological cancer, Risk factors

## Abstract

To investigate factors influencing cervical cancer and precancerous lesions by collecting and analyzing clinical data and pathological results from 2,998 patients undergoing cervical biopsy; Pathological results and clinical data of 2,998 patients who underwent cervical biopsy at the gynecology outpatient clinic of a tertiary hospital in Hangzhou (January 2022–January 2023) were collected. Univariate analysis and multivariate unconditional logistic regression models were used to analyze relevant influencing factors; The results of univariate and multivariate analyses showed that cervical lesions were related to Human Papillomavirus (HPV) infection and the number of deliveries (*P* < 0.05) but were not related to age, number of pregnancies, number of abortions, use of condoms for contraception, and smoking (*P* > 0.05); Cervical cancer and precancerous lesions are associated with HPV infection and number of deliveries. It is imperative to implement a series of prevention and treatment measures to inhibit the onset and progression of cervical diseases, thereby reducing their incidence and mortality rates.

## Introduction

Cervical cancer (CC) ranks as the fourth most common malignancy and the leading gynecological cancer globally among women, with over 600,000 new cases and 350,000 cancer-related deaths annually^1,2^, posing a severe threat to women’s health. Cervical intraepithelial neoplasia (CIN) represents a group of precancerous lesions strongly associated with invasive cervical cancer. Based on severity, CIN is classified into CIN I (mild dysplasia), CIN II (moderate dysplasia), and CIN III(severe dysplasia/carcinoma in situ). The updated classification system categorizes cervical precancers into low-grade squamous intraepithelial lesions (LSIL) and high-grade squamous intraepithelial lesions (HSIL). According to P16 immunohistochemical staining results, LSIL encompasses CIN I and CIN II [P16(-)], HSIL includes CIN II [P16(+)] and CIN III^3^. Both classification systems provide critical diagnostic and therapeutic guidance for clinical management. Persistent human papillomavirus (HPV) infection is a critical etiological factor in the development of cervical epithelial lesions and carcinogenesis^4^. Statistics show that the probability of HPV infection in a woman’s lifetime is as high as 80%, however, most are transient infections, with approximately 90% of HPV infections cleared by the immune system^5^. Only 5–10% develop persistent infections, and only 2–3% of HPV infections progress to cervical cancer^6^. Consequently, HPV infection may lead to cervical lesions but does not necessarily lead to cancer. Cervical carcinogenesis constitutes a multifactorial, multistage, and complex progressive process. Analysis of influencing factors for cervical lesions necessitates comprehensive consideration of HPV infection alongside other synergistic elements that may either elevate or mitigate the risk of lesion development and progression. By implementing targeted prevention and intervention strategies, the incidence and mortality rates of cervical cancer can be effectively reduced. This study investigates factors associated with cervical cancer and precancerous lesions through systematic collection and analysis of clinical data and pathological results from 2,998 patients undergoing cervical biopsy, as detailed below.

## Data and methods

### General data

Pathological results and clinical data were collected from 2,998 patients who underwent cervical biopsy at the gynecology outpatient clinic of a tertiary hospital in Hangzhou between January 2022 and January 2023. Inclusion criteria comprised: (1) Patients receiving colposcopy-guided biopsy or cervical conization at our institution; (2) No history of total hysterectomy; (3) No prior pelvic radiotherapy or chemotherapy. Exclusion criteria included: (1) Pregnant women; (2) History of total hysterectomy; (3) Previous pelvic radiotherapy or chemotherapy; (4) Concurrent malignancies at other sites; (5) Incomplete clinical information. This study protocol was approved by the hospital’s Institutional Ethics Committee(batch number ZN-2024353-01).

### Methods

#### Pathological grading and grouping

(1) Pathological Grading: (a) No dysplasia; (b) Low-grade squamous intraepithelial lesion (LSIL) of the cervix; (c) High-grade squamous intraepithelial lesion (HSIL) of the cervix; (d) Cervical squamous cell carcinoma (CSCC). (2) Grouping Criteria: Patients were stratified into two groups based on cervical tissue lesion severity: (a) LSIL or lower group(encompassing no dysplasia and LSIL); (b) HSIL or higher group(including HSIL and invasive carcinoma).

#### Observational indicators

Clinical data collection encompassed patient demographics, lifestyle factors, marital and reproductive history, contraceptive methods, HPV vaccination status, pre-biopsy HPV-DNA test results (manufacturer: Yaneng Bio-technology (Shenzhen) Co., Ltd., China), Thinprep cytologic test (TCT) results before biopsy, pathological findings of cervical biopsies, and follow-up HPV/TCT retest outcomes. Univariate analysis and multivariate unconditional logistic regression models were employed to identify influencing factors associated with cervical cancer and precancerous lesions.​.

#### Statistical methods

Statistical analyses were performed using SPSS version 25.0. Categorical data are expressed as the number of cases (percentage). The chi-square (x^2) test was used for comparisons. Multivariate analysis was conducted using multivariate unconditional logistic regression. Statistical significance was set at *P* < 0.05.

## Results

### Clinical/Epidemiological characteristics

**Fig. 1:**
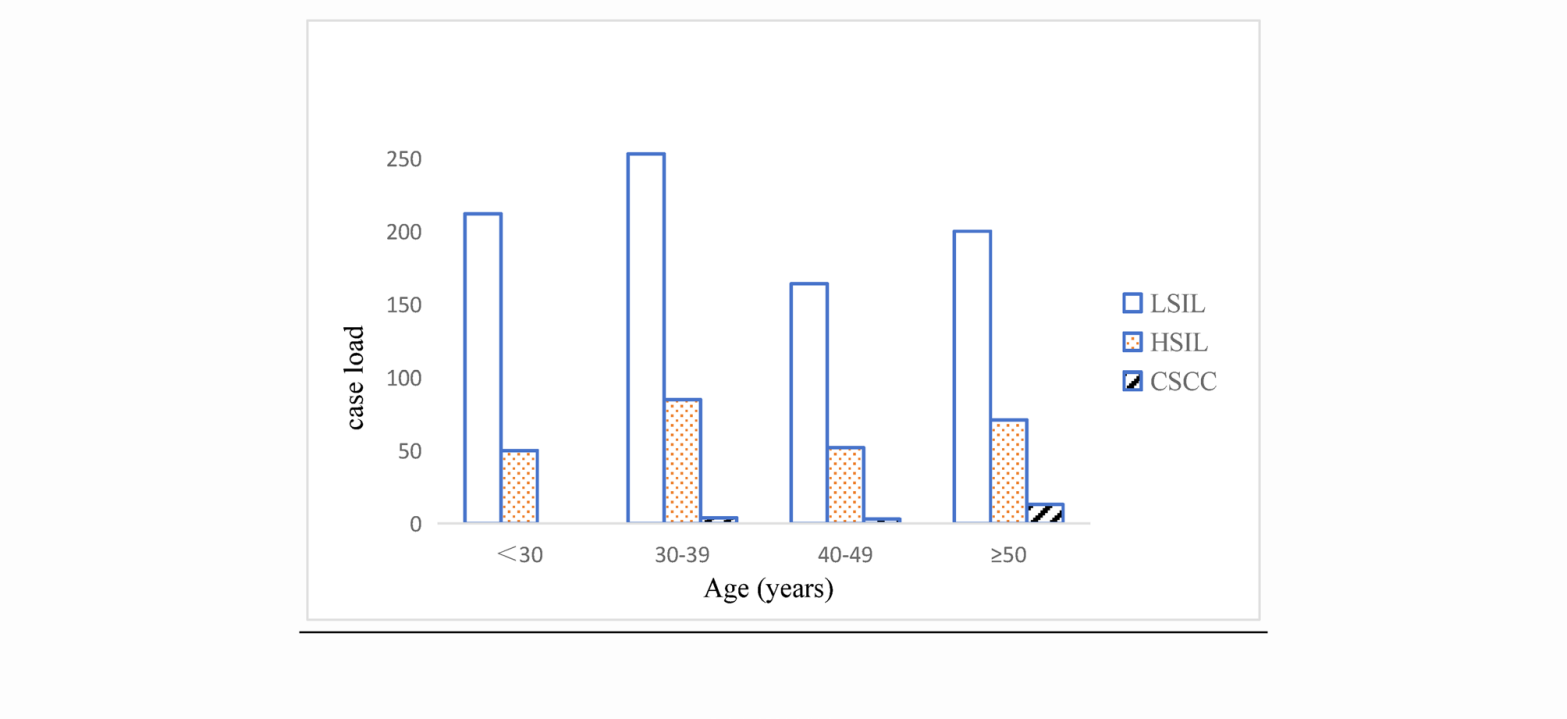
Age distribution of cervical lesions.

This study enrolled 2,998 patients with cervical lesions aged 16–76 years, comprising 1,891 cases without dysplasia, 1,087 precancerous lesions (829 LSIL and 258 HSIL), and 20 cervical cancer cases. Intergroup age comparisons showed no statistically significant difference (*p* > 0.05), indicating comparable baseline characteristics. Among 829 LSIL patients, 212 (25.6%) were aged < 30 years, 253 (30.5%) were aged 30–39, 164 (19.8%) were aged 40–49, and 200 (24.1%) were aged ≥ 50. For 258 HSIL patients: 50 (19.4%) < 30 years, 85 (32.9%) 30–39, 52 (20.2%) 40–49, and 71 (27.5%) ≥ 50. All 20 cervical cancer cases occurred in patients ≥ 30 years: 4 (20.0%) at 30–39, 3 (15.0%) at 40–49, and 13 (65.0%) at ≥ 50. Precancerous and cancerous lesions demonstrated a trend toward younger age onset, with the majority concentrated in the 30–39 and ≥ 50 age groups(Fig. 1).

Among 829 patients with cervical LSIL, 768 were infected with high-risk HPV (including 219 cases of HPV16/18 subtypes). For 258 HSIL patients, 246 had high-risk HPV infections (131 HPV16/18 cases). Of 20 cervical cancer patients, 19 tested positive for high-risk HPV (13 HPV16/18 cases). High-risk HPV was detected in approximately 92.6% of LSIL, 95.3% of HSIL, and 95.0% of cervical cancer cases. HPV16/18 infections were detected in approximately 26.4% of LSIL, 50.8% of HSIL, and 65.0% of cervical cancer cases. Among high-risk HPV infections contributing to cervical precancerous lesions and cancer, HPV16/18 subtypes accounted for 35.1%. Additionally, only 412 of 2,998 patients (13.7%) received HPV vaccination (Table 1).

**Table 1:** Cervical lesions, HPV infection and HPV vaccination status [n(%)].

Item	No dysplasia *n* = 1891	LSIL *n* = 829	HSIL *n* = 258	CSCC *n* = 20	Total *n* = 2998
HPV infected cases	1775 (93.9)	802 (96.7)	247 (95.7)	19 (95.0)	2843 (94.8)
High-risk HPV infected	1629 (86.1)	768 (92.6)	246 (95.3)	19 (95.0)	2662 (88.8)
HPV16/18 type infected cases	499 (26.4)	219 (26.4)	131 (50.8)	13 (65.0)	862 (28.8)
HPV vaccinated cases	263 (13.9)	116 (14.0)	31 (12.0)	2 (10.0)	412 (13.7)

### Correlation analysis of influencing factors of cervical cancer and precancerous lesions

#### Univariate analysis

Univariate analysis showed that cervical cancer and precancerous lesions were associated with HPV infection and the number of deliveries (*P* < 0.05) (Table 2).

**Table 2:** Univariate analysis of factors influencing cervical cancer and precancerous lesions.

Item	Cervical tissue lesion severity		Exp (B)	*P*
	LSIL and below *n* = 2720	HSIL and above *n* = 278		
Age (-x±s, years)	57 ± 19.89	44 ± 13.30	1.003	0.643
HPV infection cases [n(%)]			2.199	0.000
HPV negative	143(5.3)	12(4.3)		
HPV low-risk type	180(6.6)	1(0.4)		
Other high-risk types	1679(61.7)	121(43.5)		
HPV16/18 types	718(26.4)	144(51.8)		
Pregnancy times [n(%)]			0.888	0.453
> 2 times	1517(55.8)	147(52.9)		
≤ 2 times	1203(44.2)	131(47.1)		
Delivery times [n(%)]			2.172	0.002
> 2 times	2590(95.2)	252(90.6)		
≤ 2 times	130(4.8)	26(9.4)		
Abortion times [n(%)]			1.440	0.077
> 2 times	2421(89.0)	238(85.6)		
≤ 2 times	299(11.0)	40(14.4)		
Use of condoms for contraception [n(%)]			0.899	0.490
Yes	1149(42.2)	107(38.5)		
No	1571(57.8)	171(61.5)		
Smoking [n(%)]			1.715	0.208
Yes	42(1.5)	7(2.5)		
No	2678(98.5)	271(97.5)		

#### Multivariate analysis

**Fig. 2:**
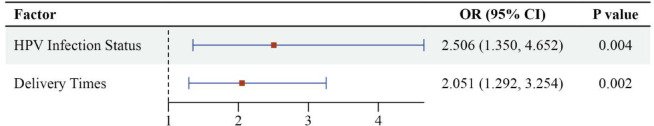
Forest map of factors affecting cervical cancer and precancerous lesions.

Using multivariate unconditional logistic regression analysis with cervical tissue lesion severity (HSIL or higher vs. LSIL or lower) as the dependent variable, and HPV infection status (coding scheme: HPV-negative = 0; low-risk HPV infection = 1; other high-risk HPV infection = 2; HPV16/18 infection = 3) and delivery times (coding scheme: >2 deliveries = 1, ≤ 2 deliveries = 0) as independent variables. The multivariate unconditional logistic regression analysis results showed that cervical cancer and precancerous lesions were associated with HPV infection and the number of deliveries (*P* < 0.05). High-risk HPV infection exerted a substantial impact on HSIL + progression, while multiple deliveries increase the risk of cervical cancer and precancerous lesions (Table 3; Fig. 2).

**Table 3:** Multivariate unconditional logistic regression analysis of factors influencing cervical cancer and precancerous lesions.

Factor	B	Wald	*P*	Exp (B)	95% CI for Exp (B)	
					Lower limit	Upper limit
HPV infection status Negative Low-risk type Other high-risk types HPV16/18 type	0.919	8.467	0.004	2.506	1.350	4.652
Delivery times > 2 times ≤ 2 times	0.718	9.286	0.002	2.051	1.292	3.254

### Sensitivity analysis of HPV detection combined with TCT screening

For pathological diagnoses of LSIL or less severe lesions, the results of colposcopy-guided biopsy are classified as negative; if HSIL or more severe lesions are present, they are classified as positive. When TCT screening detects HSIL or higher-grade lesions, it is considered positive; all other cytological examination results are deemed negative. HPV testing showing high-risk infection was defined as positive, with other results negative; co-testing positivity required either HPV or TCT positive. Results demonstrated that standalone TCT screening exhibited a sensitivity of 19.4% for detecting HSIL + lesions (Table 4), whereas HPV co-testing achieved 97.5% sensitivity (Table 5).

**Table 4:** TCT screening and pathological test results [n(%)].

TCT results	Pathological test results		
	Negative	Positive	Total
Negative	2698(90.0)	22(0.7)	2720(90.7)
Positive	224(7.5)	54(1.8)	278(9.3)
Total	2922(97.5)	76(2.5)	2998(100.0)

**Table 5:** Results of HPV combined with TCT and pathological test results [n(%)].

HPV combined with TCT results	Pathological test results		
	Negative	Positive	Total
Negative	318(10.6)	2402(80.1)	2720(90.7)
Positive	7(0.2)	271(9.0)	278(9.3)
Total	325(10.8)	2673(89.2)	2998(100.0)

### Follow-up status

**Fig. 3:**
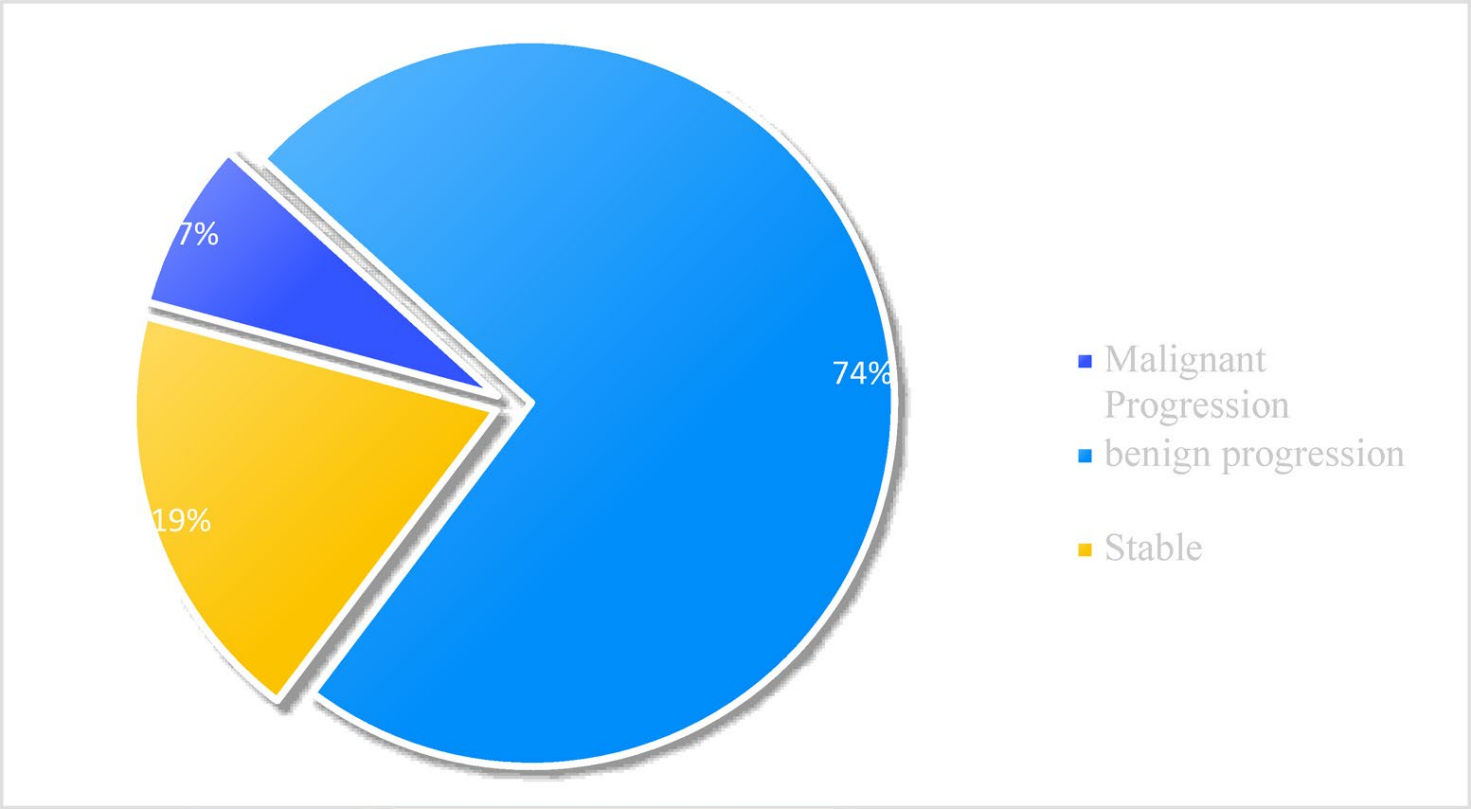
Follow-up after cervical LSIL biopsy.

**Fig. 4:**
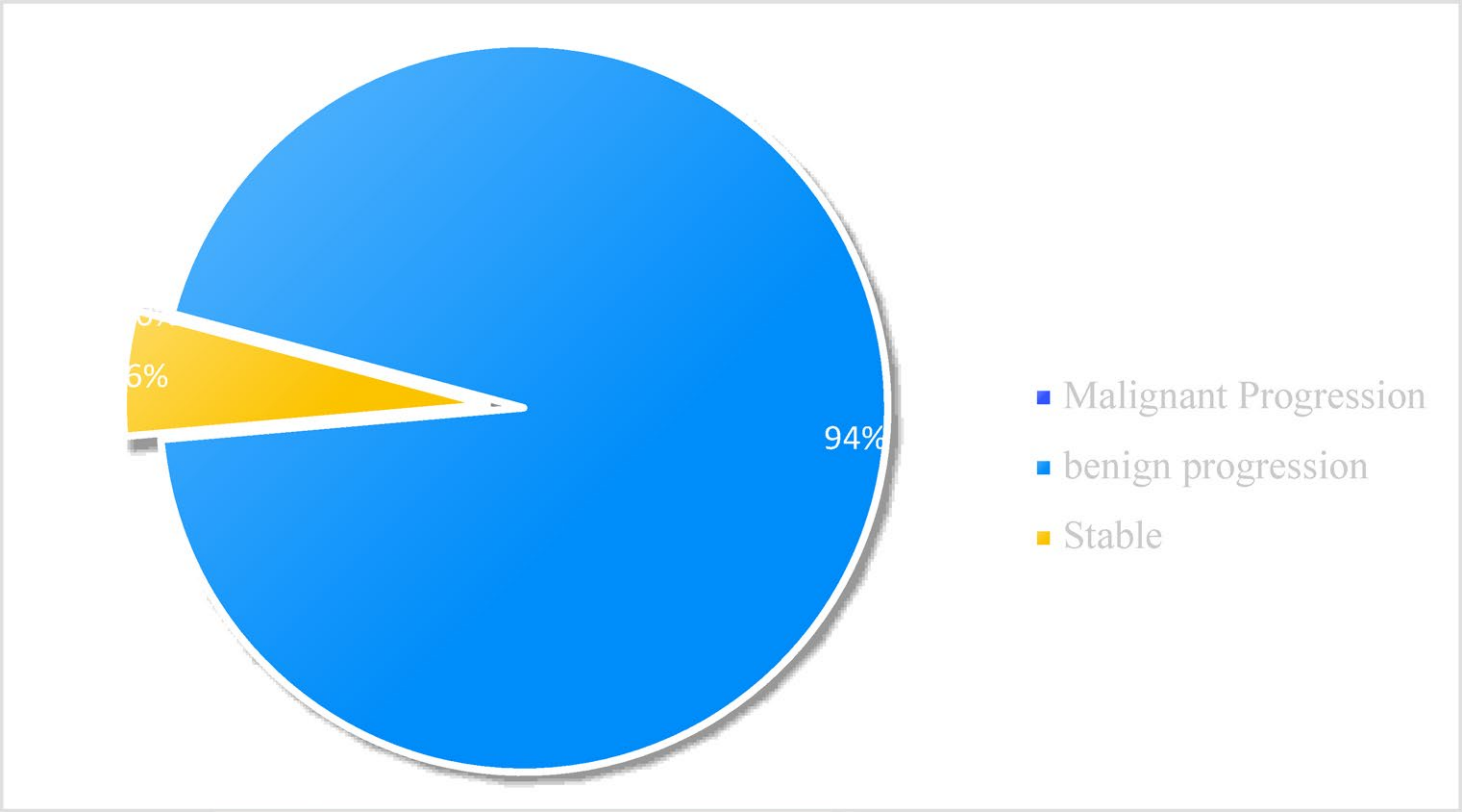
Follow-up after cervical HSIL biopsy.

A 1-year follow-up study tracking cervical lesion outcomes was conducted in patients post-biopsy, with 150 cases completing HPV + TCT retests. Among these, 61 patients initially without dysplasia showed 4 cases of malignant progression (including SIL and CSCC), 57 stable; 53 LSIL patients had 4 malignant progressions (HSIL/CSCC), 39 benign progression(no dysplasia), and 10 stable (Fig. 3); 36 HSIL patients exhibited 0 malignant progression(CSCC), 34 benign progression(LSIL/no dysplasia), and 2 stable (Fig. 4). Collectively, cervical abnormalities demonstrated significantly fewer malignant progressions than benign progressions post-biopsy.

## Discussion

According to the data report released by the National Cancer Center, in 2022, China recorded 151,000 new cases of cervical cancer, with an incidence rate of 13.8 per 100,000, and 56,000 deaths related to cervical cancer, resulting in a mortality rate of 4.5 per 100,000^7^. Therefore, efforts in the prevention and control of cervical cancer still require reinforcement. This study aims to investigate the influencing factors of cervical cancer and its precancerous lesions, propose targeted recommendations, prevent the onset of cervical lesions, and delay their progression.

Currently, the diagnosis of cervical squamous intraepithelial lesions (SIL) and invasive cervical cancer primarily relies on the “three-step procedure,” which includes cervical cytology/HPV testing, colposcopy, and cervical biopsy. This study found that cytological examination has low sensitivity; however, combining cervical cytology with HPV testing can enhance the sensitivity for detecting cervical cancer and high-grade squamous intraepithelial lesions (HSIL). Where economically feasible, it is recommended to use combined cervical HPV testing and cytology to improve disease detection rates. Among patients meeting the indications who underwent early surgery, follow-up observations revealed more cases of benign progression than malignant progression of cervical lesions. This not only indicates that cervical biopsy serves a dual role in both diagnosis and treatment, but also suggests that timely intervention for precancerous lesions can delay disease progression.

Cervical carcinogenesis is a multifactorial, multi-stage, and complex progressive process. HPV infection is one of the independent risk factors for cervical lesions, with individuals infected with HPV facing a 250-fold higher risk of developing cervical cancer compared to those without infection^8^. Additionally, the onset and progression of cervical lesions may also be associated with numerous other factors, including age, multiple sexual partners, early sexual debut, sexually transmitted infections (STIs), smoking, oral contraceptive use, genetic predisposition, and immune status.

This study found that cervical precancerous lesions and carcinogenesis are associated with HPV infection, particularly high-risk HPV (hrHPV) infection. High-risk HPV infections were detected in approximately 92.6% of low-grade squamous intraepithelial lesion (LSIL) cases, 95.3% of high-grade squamous intraepithelial lesion (HSIL) cases, and 95.0% of cervical cancer patients. HPV is a non-enveloped spherical DNA virus, and persistent HPV infection is a critical cause of cervical intraepithelial lesions and carcinogenesis^9^. HPV is primarily transmitted through sexual contact; thus, having multiple sexual partners, unprotected sexual intercourse, and sexually transmitted infections (STIs) can all increase the risk of HPV acquisition. Multiple sexual partnerships not only elevate the risk of HPV transmission and cervical cancer^10^, but may also predispose individuals to other STIs, such as HIV, which further heightens the risk of cervical cancer and precancerous lesions^11^. Additionally, HPV16/18 infections were detected in approximately 26.4% of LSIL, 50.8% of HSIL, and 65.0% of cervical cancer cases. HPV16/18 accounted for 35.1% of hrHPV infections associated with cervical precancerous lesions and carcinogenesis. On the one hand, this underscores the necessity of vaginal colposcopy and biopsy for women infected with HPV16/18 to facilitate early detection of lesions; on the other hand, it emphasizes that the impact of other hrHPV subtypes on cervical lesions should not be overlooked. Given that HPV subtype distributions vary across different regions, it is recommended to implement genotyping HPV nucleic acid testing and strengthen management for patients infected with regionally prevalent hrHPV subtypes.

This study found that cervical precancerous lesions and cervical cancer are associated with the number of deliveries, with patients who have undergone multiple deliveries exhibiting an elevated risk of cervical cancer and precancerous lesions. Numerous studies have also indicated that cervical lesions are linked to reproductive factors, including age at first pregnancy, number of abortions, number of deliveries, and number of pregnancies. A younger age at first pregnancy increases the risk of invasive cervical cancer and cervical carcinoma in situ^12^, which may be associated with the higher HPV infection rate among women who become sexually active at a young age. Increased numbers of deliveries and abortions lead to repeated damage to cervical tissue, weakening the cervical barrier function. Moreover, the process of cervical tissue repair following injury is accompanied by cellular metaplasia, which facilitates HPV invasion and thus accelerates the progression of cervical lesions^6^. Muñoz N et al. found that high parity increases the risk of squamous cell carcinoma of the cervix in HPV-positive women: compared with nulliparous women, the odds ratio (OR) was 3.8 for women with ≥ 7 term pregnancies, and 2.3 when compared with women with 1–2 term pregnancies^13^. Changes in blood estrogen and progesterone levels during pregnancy lead to alterations in the squamous-columnar epithelial junction (transformation zone) of the cervix. Ectopy of the columnar epithelium to the peripheral cervix (cervical ectopy) begins in early pregnancy and becomes more pronounced in mid-to-late pregnancy. Squamous metaplasia within the transformation zone also increases during gestation, reaching its peak in late pregnancy. Consequently, multiple pregnancies may elevate the risk of cervical cancer, as they maintain the transformation zone outside the cervix for an extended period, thereby facilitating direct exposure to HPV and other cofactors^13^. Additionally, studies^14,15^ have reported that long-term use of oral contraceptives (≥ 5 years) increases the risk of cervical neoplasia. Women with more than 5 term pregnancies and those who have used oral contraceptives for over 5 years face a nearly 12-fold elevated risk of squamous cell carcinoma (SCC). Thus, it is advised that women marry and bear children at an appropriate age, select suitable contraceptive methods, and reduce the frequency of abortions.

This study found that cervical precancerous lesions and carcinogenesis are not associated with increasing age; however, cervical lesions exhibit a trend of younger onset, predominantly affecting women aged 30–39 years and those aged ≥ 50 years. A large-scale study^16^ revealed that HPV detection rates show two peak periods with advancing age: one in the 17–24-year-old group and another in the 40–45-year-old group. The higher detection rate in the former may be attributed to more frequent sexual activity, earlier cervical exposure, and an immature immune system that renders young women more susceptible to HPV infection^17^. The second peak in the latter group may be linked to lower estrogen levels, reduced immune function, and heightened susceptibility to HPV infection coupled with diminished viral clearance capacity^18^. Health education for women in these age groups should be strengthened, encouraging regular cervical cancer screening to enable early detection, diagnosis, and treatment. Regarding the trend of younger-onset cervical lesions, it is considered partially related to early sexual activity; thus, emphasis should be placed on adolescent sexual education.

This study found that cervical precancerous lesions and carcinogenesis are not associated with condom use. However, R.L.W. et al. reported that the prevalence of genital HPV infection was 37.8% among women whose partners used condoms consistently during every sexual intercourse, compared to 89.3% among those whose partners used condoms less than 5% of the time. Furthermore, women whose partners did not use condoms or used them inconsistently exhibited a higher detection rate of squamous intraepithelial lesions (SIL)^19^. The use of condoms or dental dams can act as a physical barrier, thereby reducing HPV transmission^20^ and lowering the risk of cervical lesion progression. Therefore, condoms should be used consistently throughout both sexual intercourse and non-coital sexual activities.

This study did not indicate an association between cervical precancerous lesions/carcinogenesis and smoking. However, active smoking is uncommon among women, with most cases involving passive smoking, making data collection challenging. Thus, the possibility that smoking increases the risk of HPV infection and cervical precancerous lesions in women cannot be ruled out. Research by Hu Shangying et al.^21^ revealed that women who smoke actively have a 1.45-fold higher risk of HPV infection and a 1.89-fold increased risk of cervical intraepithelial neoplasia grade 2 or higher (CIN2+) compared to non-smokers. Women exposed to passive smoking have a 1.11-fold higher risk of HPV infection than those not exposed to passive smoking. Additionally, both active and passive smoking exhibit a synergistic effect on the occurrence of HPV infection and CIN2+. The mechanism by which smoking contributes to cervical lesions remains unclear. It may be related to soluble carcinogens in cigarette smoke—such as nicotine, cotinine, and NNK—which interfere with cervical epithelial metaplasia, induce oncogenic gene alterations, and disrupt cervical mucosal immunity^22,23^. Therefore, it is necessary to strengthen relevant health education, enforce tobacco control policies more rigorously, and reduce the prevalence of both active and passive smoking.

HPV vaccination serves as primary prevention against cervical cancer and is an effective measure for its prevention. HPV vaccines exhibit immunogenicity, safety, and high efficacy. These vaccines include bivalent vaccines (targeting HPV16 and 18), tetravalent vaccines(targeting HPV16, 18, 6, and 11), and nine-valent vaccines (targeting HPV6, 11, 16, 18, 31, 33, 45, 52, and 58). All are based on HPV virus-like particles(VLPs) as antigens, which stimulate the body to produce antibodies and exert their preventive effects. The safety of these vaccines lies in the fact that VLPs do not contain viral DNA, rendering them non-infectious; no serious adverse events associated with the vaccines have been identified to date. HPV vaccines demonstrate strong protective efficacy against HPV infection in women, and numerous studies have confirmed their effectiveness in preventing HPV infection and the development of cervical lesions^24–26^. Notably, reports have indicated that vaccinating individuals already infected with HPV16/18 does not confer therapeutic benefits and may even increase the risk of cervical lesions^27^. Therefore, further research is needed to evaluate the benefits of HPV vaccination for individuals with existing HPV infections to determine whether it should be recommended. Among the 2,998 cases in this study, only 13.7% had received HPV vaccination. This low coverage may be influenced by multiple factors, including vaccine cost, economic and health conditions, education levels, and awareness of the vaccine. Therefore, measures such as expanding HPV vaccination awareness campaigns, adjusting vaccine pricing, and developing appropriate immunization schedules could help increase vaccination coverage and reduce the incidence of cervical cancer.

The innovation points of this study are as follows: (1) By analyzing the epidemiological characteristics and influencing factors of cervical lesions, targeted health education and preventive measures are proposed; (2) Combined HPV + TCT screening improves the detection rate of cervical lesions of HSIL (High-Grade Squamous Intraepithelial Lesion) and above; (3) For patients meeting the indications, early cervical biopsy treatment can delay disease progression. The original intention and ultimate goal of the study are to prevent the occurrence of diseases and delay their progression.

This study has certain limitations: (1) The analysis of influencing factors for cervical lesions was conducted retrospectively, which precludes the rigorous control of variables as in experimental studies. Additionally, due to factors such as population mobility and unplanned pregnancies, follow-up was challenging, resulting in a relatively high loss to follow-up rate. To address this, the necessity of follow-up visits should be emphasized during patient consultations, detailed long-term treatment plans should be developed, and close monitoring of disease progression in patients should be prioritized. (2) Due to the small number of individuals who received HPV vaccination, varied vaccine types, and different vaccination timings, the vaccination status could not be temporarily included in the comparative analysis. However, with the increasing popularity of HPV vaccination and the enhancement of public health awareness, an increasing number of young people have started to receive HPV vaccination. Future studies may further explore the associations between different age groups, administration of different vaccines, vaccination timing, and the occurrence/progression of cervical lesions, thereby evaluating the preventive efficacy of HPV vaccines against cervical cancer. (3) The study results differed significantly from initial expectations. Subsequent research could incorporate other potential risk factors, such as education level, HIV testing history, history of genital warts, history of autoimmune diseases, history of cervical intraepithelial neoplasia(CIN), and age at first sexual intercourse. By exploring these additional factors and developing a comprehensive risk model, the findings could gain greater clinical relevance.

In summary, cervical precancerous lesions and carcinogenesis are associated with HPV infection, particularly hrHPV infection. It is recommended to implement genotyping HPV nucleic acid testing, emphasize infections with hrHPV types other than 16/18, and promote combined HPV-TCT screening to enhance the sensitivity of detecting HSIL and above. Timely management of HSIL and above lesions can delay disease progression. The risk of cervical precancerous lesions and carcinogenesis increases with the number of deliveries; thus, promoting childbearing at an appropriate age, selecting suitable contraceptive methods, and reducing the frequency of abortions are advisable. Cervical cancer and precancerous lesions predominantly affect women aged 30–39 years and those aged ≥ 50 years, with a trend toward younger onset. Health education for women in these age groups and adolescent sexual education should be strengthened, and women of appropriate age with a history of sexual activity should be encouraged to undergo regular cervical cancer screening. Through these measures, the occurrence and progression of cervical diseases can be prevented, ultimately reducing the incidence and mortality rates of the disease.

## Data Availability

The date underlying this article is shared upon reasonable request with the corresponding author.
